# Childhood trauma cortisol and immune cell glucocorticoid transcript levels are associated with increased risk for suicidality in adolescence

**DOI:** 10.1038/s41380-025-02923-3

**Published:** 2025-02-24

**Authors:** Tanya Goltser-Dubner, Fortu Benarroch, Michal Lavon, Reaan Amer, Laura Canetti, Ruth Giesser, Ella Kianski, Josef Martin, Dalya Pevzner, Pnina Blum Weinberg, Amichai Ben-Ari, Moriah Bar-Nitsan, Shaked Alon, Shai Yshai, Amit Lotan, Esti Galili-Weisstub, Ronen Segman, Amit Shalev

**Affiliations:** 1 Molecular Psychiatry Laboratory, Hadassah Medical Organization and Faculty of Medicine, Hebrew University of Jerusalem, Jerusalem, Israel; 2 The Herman-Danna Department of Child and Adolescent Psychiatry, Hadassah Medical Organization and Faculty of Medicine, Hebrew University of Jerusalem, Jerusalem, Israel; 3 Department of Psychology, Hebrew University of Jerusalem, Jerusalem, Israel; 4 The Donald Cohen Child and Adolescent Psychiatry Department, Eitanim Psychiatric Hospital, The Jerusalem Mental Health Center, Jerusalem, Israel; 5 Department of Behavioral Sciences, Ariel University, Ariel, Israel

**Keywords:** Prognostic markers, Molecular biology

## Abstract

Rising adolescent suicide rates present a growing unmet need. Childhood trauma (CT) has been associated with altered cortisol dynamics and immune cell glucocorticoid reactivity, yet their additive longer-term contributions to later suicide outcomes are less clear. The current study compared CT scores, resting salivary free cortisol and mononuclear cell gene expression levels of the nuclear receptor, subfamily 3, member 1 (NR3C1) coding the glucocorticoid receptor, and its co-chaperons FKBP prolyl isomerase 5 (FKBP5) and KIT Ligand (KITLG), between a cohort of adolescents presenting with a suicidal crisis requiring hospital treatment, and matched healthy controls. Childhood trauma scores and glucocorticoid measures were significantly altered among suicidal adolescents, and CT scores correlated with mononuclear cell glucocorticoid transcripts. Both CT scores and glucocorticoid measures explained substantial additive portions of the variance in adolescent suicidality. Long-term perturbations in cortisol dynamics and immune cell glucocorticoid response elements denote dysregulated immune stress reactivity, and may possess value in prediction and point to modifiable-risk factors in prevention of clinically significant suicidality during the brittle period of adolescence, years after childhood trauma exposure.

## Introduction

Addressing the alarming rise in suicide rates among younger age groups [1], necessitates a better understanding of the environmental and biological interplay contributing to these complex behavioral propensities [2, 3]. Exposure to trauma in childhood has been associated with persisting abnormalities in glucocorticoid dynamics [4–6], immune reactivity to cortisol [7], and a pro-inflammatory state [8, 9]. Childhood trauma (CT) has been repeatedly demonstrated to constitute a major risk factor for later psychopathology [2], and reduced life span [10], in part due to increased risk for suicide [11]. Altered cortisol and gene expression levels of NR3C1 and FKBP5 have been previously associated with both childhood trauma and suicide risk as discussed in detail below. Associations of KITLG gene expression levels with childhood trauma and suicidality have not been explored to date. The association of persisting endocrine immune changes with early trauma exposure and suicide risk later in life require further delineation. In the current study, we demonstrate that adolescents presenting with a significant suicidal crisis requiring hospital care, have higher CT scores as well as reduced resting morning salivary cortisol and altered mononuclear cell expression of genes encoding the interactive glucocorticoid response elements, NR3C1, FKBP5, and KITLG. Early trauma exposure and glucocorticoid indices each contributed to explain significant parts of the variance in adolescent suicidal risk.

## Materials and methods

### Clinical procedure

Adolescents requiring care at the Herman Dana Pediatric Psychiatry department at the Hadassah Medical Center following a significant suicidal crisis, were approached to participate in the current study. Suicidal adolescents were compared with healthy adolescents evaluated to exclude medical or psychiatric illness. Inclusion criteria included: adolescents aged 12–18 years presenting to the in-patient unit, the daycare unit, or the crisis intervention clinic with recent suicidal ideation (SI) with plan or intent and/or recent suicide attempt (within the last month). We defined a suicide attempt as per the Columbia Clinical Algorithm for Suicide Assessment (C-CASA) [12], as “self-destructive behavior with inferred or stated intent to die”. For subjects under 18 years, patients and parents or guardians with legal custody had to both be available and able to provide informed consent for participation in the study. Exclusion criteria included mania, active psychosis, autism, substance use disorder, intellectual disability, serious physical injuries, head trauma, or medical ailments. Matched healthy controls were included if they had no current or past psychiatric or medical diagnoses upon clinical evaluation. Consenting subjects underwent structured clinical assessment by the study psychiatrist (A.S.) using the Kiddie-Schedule for Affective Disorders and Schizophrenia (K-SADS) Semi-structured clinical diagnostic interview for DSM-V to diagnose/exclude axis I psychiatric disorders. The Childhood Trauma Questionnaire (CTQ) [13] was used to rate childhood adversity exposure. The CTQ score increases with cumulative exposure to adverse events experienced as traumatic over childhood and adolescence. The Patient Health Questionnaire-9 (PHQ-9) [14] was used to rate current depression severity. All subjects underwent an extensive clinical evaluation.

#### Saliva and Blood mononuclear cell collection and separation

Morning saliva and blood samples were collected following admission. SalivaBio oral swabs were applied according to manufacturer’s instructions (Salimetrics Carlsbad CA). Participants were instructed to avoid food, liquids, and teeth brushing, and rinse their mouth with water 10 min prior to sampling. Samples were stored at 4 °C for 24 h, centrifuged and stored at −80 °C until assaying with duplicates using ELISA (Salimetrics LLC, Carlsbad CA) as previously described [15]. Intra and inter assay variabilities were both below 10%. Venous blood sample were taken by venipuncture collected with EDTA and immediately centrifuged using Histopack gradient to separate peripheral blood mononuclear cells (PBMCs), and stored at −80 °C until processing as previously described [16]. All lab procedures were preformed blind as to sample allocation.

### Gene expression

Briefly, one microgram RNA from Monocytes was reverse transcribed using High Capacity cDNA RT kit (Applied Biosystems, Foster City, CA, USA) as per manufacturer instructions. Amplification reactions were performed in 20 µl volume: 10 µl TaqMan PCR master mix (Applied Biosystems), 2 µl cDNA, 1 µl Assays on-Demand™ (Applied Biosystems). The cDNA was quantified using real-time quantitative PCR performed in a GeneAmp 7500 Sequence Detection System (Perkin Elmer, USA), with Sequence Detection System software (Applied Biosystems, Foster City, CA, USA) using specific TaqMan^TM^ primers and probe for the NR3C1, FKBP5 and KITLG transcripts relative to 2 reference genes TUBB and GUSB with unchanged expression in mononuclear cells, according to methods previously described by us [17].

### Data-analysis

To compare resting morning salivary cortisol and peripheral blood mononuclear cell (PBMC) expression levels of the three transcripts between the Suicidal Youth (SY) and Healthy Youth (HY) groups, we conducted MANOVA and univariate ANOVA between the SY group sampled at hospital admission following a suicide attempt, compared with HY control group. We also compared CTQ scores between groups using univariate ANOVA. Demographic and clinical variables were added as co-variants to exclude confounding. Logistic regression was employed to examine the contribution of CTQ scores and glucocorticoid indices to risk of being assigned to the SY group, and linear regression was applied to examine their contribution to suicidal ideation scores in the PHQ-9. Lab and statistical analyses were done blind as to case control allocation.

### Ethics approval and consent to participate

All methods were performed in accordance with the relevant guidelines and regulations. The study was approved by the Hadassah Medical Center Intitutional Review Board (HMO -0225-19) and the Israel Ministry of Health Review Board (202013923), and all subjects, and if minors also their parents\legal guardians, signed informed consent forms.

## Results

Sixty three suicidal adolescents meeting above inclusion and exclusion criteria were prospectively ascertained following admission for a significant suicidal crisis and compared with 69 medically and psychiatrically healthy adolescent controls. Data was normally distributed. There was a significant age difference between SY (n = 63) 15.4 ± 2.1 years, and HY (n = 69) 18.6 ± 0.8 years, t = 11.4 *df* = 130, p < 0.001; and a significant gender difference between the SY, male n = 8 (12.7%) and female n = 55(87.3%), and HY, male n = 32 (46.4%) and female n = 37(53.6%) *X*^2^ = 17.6, df = 1 p < 0.001. Age and gender were not correlated with CTQ, cortisol, or gene expression levels.

**Fig. 1:**
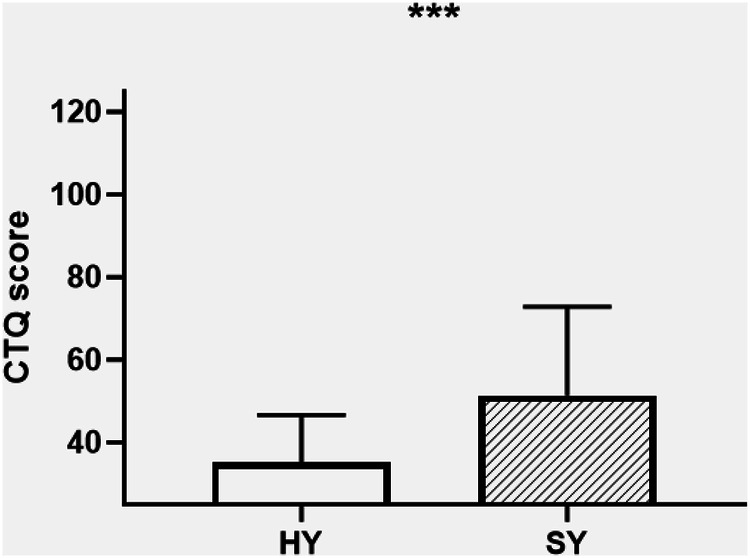
Childhood trauma scores among suicidal *vs*. healthy control adolescents. Suicidal Youth (SY, n = 62) showed significantly higher Childhood Trauma Questionnaire (CTQ) scores compared with Healthy Youth controls (HY, *n* = 53); Univariate ANOVA CTQ SY (*n* = 62) 51.9 ± 21.7 *vs*. HA (*n* = 53) 35.1 ± 11.6, F = 21.8, df = 1111, p < 0.001, *** = p ≤ 0.001.

We next compared salivary cortisol, and mononuclear cell NR3C1 FKPB5 and KITLG PBMC mRNA levels, between the 63 SY and 69 HY subjects. Univariate ANOVA including age and gender as covariates demonstrated significantly higher Childhood Trauma Questionnaire (CTQ) scores among 59 suicidal adolescents compared with 53 healthy adolescent controls: CTQ SY (n = 59) 51.4 ± 21.3 *vs*. HY (*n* = 53) 35.1 ± 11.6, F = 21.9, df = 1, p < 0.001; (Fig. 1).

**Fig. 2:**
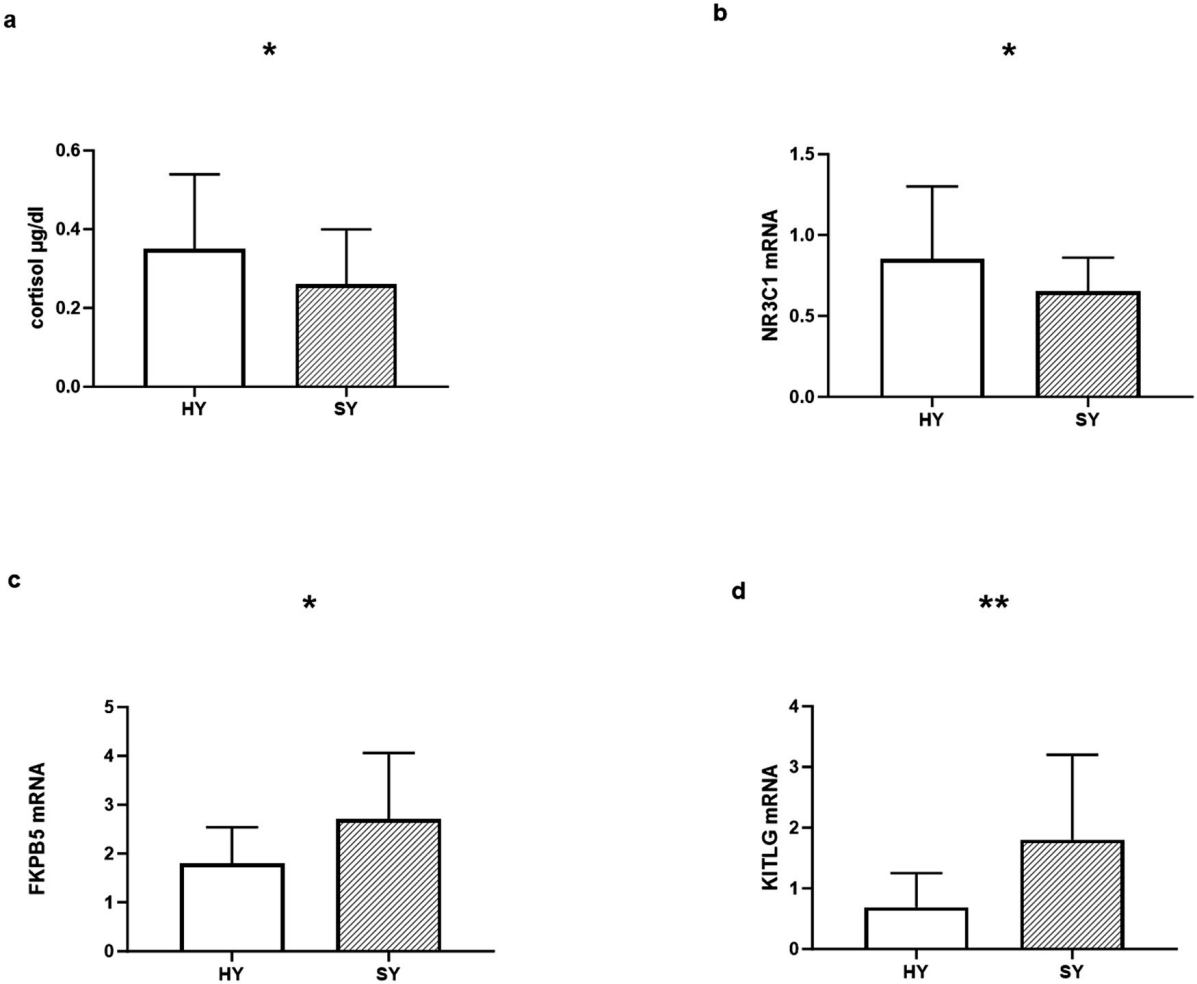
Salivary cortisol and mononuclear cell transcript levels among suicidal *vs*. healthy control adolescents. The univariate ANOVA demonstrated significantly lower resting salivary cortisol levels as well as lower PBMC NR3C1 and higher FKBP5 and KITLG transcript levels among the 63 SY, compared with 69 HY controls (Fig. 2). Cortisol SY (*n* = 63) 2.6 ± 1.4 *vs*. HY (*n* = 69) 3.5 ± 1.9, F = 4.98,df = 1132, p = 0.027 **a**; NR3C1 SY (*n* = 63)0.65 ± 0.21, *vs*. HY (*n* = 69) 0.85 ± 0.46, F = 5.70 df = 1132 p = 0.018 **b**; FKBP5 SY (*n* = 63) 2.72 ± 1.35, *vs*. HY (*n* = 69) 1.85 ± 0.75, F = 5.68, df = 1132, p = 0.019 **c**. KITLG SY (*n* = 63) 1.83 ± 1.45*vs*. HY (*n* = 69) 0.69 ± 0.56, F = 9.74, df = 1132, p = 0.002 **d**. * = p ≤ 0.05, ** = p ≤ 0.01.

The overall MANOVA including age and gender as covariates comparing cortisol and the three PBMC transcript levels exhibited a significant difference between the two groups F = 5.98, df = 4125, p < 0.001. The Holmes-Bonferroni [18] correction for multiple testing gave similar significant results (data not shown). Age and gender were not significant as co-variants. The univariate ANOVA demonstrated significantly lower resting salivary cortisol levels as well as lower PBMC, NR3C1 and FKBP5 transcript levels, and higher KITLG levels among the 63 SY, compared with 69 HY controls; Cortisol SY (*n* = 63) 0.26 ± 0.14 *vs*. HY (*n* = 69) 0.35 ± 0.19, F = 4.98, df = 1132, p = 0.027; NR3C1 SY (n = 63)0.65 ± 0.21, *vs*. HY (*n* = 69) 0.85 ± 0.46, F = 5.70, df = 1132, p = 0.018; FKBP5 SY (*n* = 63) 2.72 ± 1.35, *vs*. HY (*n* = 69) 1.85 ± 0.75, F = 5.68, df=1132, p = 0.019. KITLG SY (*n* = 63) 1.83 ± 1.45 *vs*. HY (*n* = 69) 0.69 ± 0.56, F = 9.74, df = 1132, p = 0.002; (Fig. 2).

Suicide attempts included medication over dose (n = 23), wrist cutting requiring sutures (n = 1), suffocation (n = 1) and hanging (n = 1). All subjects underwent psychiatric evaluations in the aftermath of the suicide attempt and were offered psychotherapy intervention. Forty-four of the SY subjects further required psychiatric medication treatment and all were still medicated at the time of blood sampling follow-up evaluation. Fifty six of the SY subjects were also diagnosed with comorbid psychiatric diagnoses: 35 had comorbid major depression, eight had anxiety disorders including GAD OCD PTSD and social phobia, six had eating disorders, and seven had other disorders. CTQ, salivary cortisol and mononuclear cell gene expression levels of the three PBMC transcripts NR3C1, FKBP5 and KITLG, did not correlate with age nor showed significant mean differences between males and females among the entire sample, nor showed significant differences between the 35 SY subjects with MDD and those without MDD, nor between the 56 SY with any comorbid psychiatric diagnoses and those without such diagnoses. Of the 44 SY subjects receiving medications at the time of blood sampling 35 received a selective serotonin reuptake inhibitor (SRI), 28 received a low dose anti-psychotic and 2 received a benzodiazepine. There was no significant difference for any of the above between the SY subjects who have not received medications and the 44 SY receiving medications, nor between the 35 receiving an SRI nor the 28 receiving an antipsychotic. Nor was there a significant difference between the 25 SY with suicide attempt during the past month and those without a recent attempt (data not shown). Childhood trauma scores were significantly correlated with mononuclear cell NR3C1 (n = 112, r = −0.196, p = 0.038), FKBP5 (n = 112, r = 0.218, p = 0.021), and KITLG (n = 112, r = 0.228, p = 0.016) transcript levels among the entire sample.

### Logistic regression

Logistic regression analyses were applied to further evaluate the potential contribution of CTQ as well as the potential additive contributions of glucocorticoid indices to SY vs. HY group assignment. When only the CTQ score was entered in the regression it contributed Nagelkerke R^2^ = 26.5% of the variance in the risk of being assigned to the SY group designation (n = 112) (Table 1).

**Table 1:** Suicidal adolescents group assignment variance explained by childhood trauma score.

Variable	B	SE	Wald	df	sig
CTQ	0.067	0.017	15.386	1	0.001

When only CTQ score is entered into the logistic regression model, it correctly classified 43 of 53 HY cases and 36 of 59 SY cases resulting in a 70.5% correct classification rate (Table 2).

**Table 2:** Correct classification rate of suicidal adolescents by Childhood trauma score.

	Healthy adolescents	Suicidal adolescents	Percentage Correct
Healthy adolescents	43	10	81.1%
Suicide adolescents	23	36	61.0%
Overall Percentage			70.5%

Morning cortisol levels contributed significantly to the classification when added to CTQ scores (overall Nagelkerke R Square rose to 0.32; with a significant contribution of cortisol p value 0.023), as well as when NR3C1 and FKBP5 gene expression levels were added to the model (overall Nagelkerke R Square 0.51 cortisol p value 0.02). However, following the introduction of KITLG into the regression model, the overall Nagelkerke R Square rose to 0.67 and the p value for cortisol fell to become insignificant, with the variance explained by KITLG also accounting for all the variance previously explained by cortisol. The CTQ score combined with mononuclear cell gene expression levels of NR3C1, FKBP5 and KITLG, thus contributed 67% of the variance in the risk of being assigned to the SY group designation (Table 3 and Supplementary Fig. 1).

**Table 3:** Suicidal adolescents group assignment variance explained by childhood trauma score and glucocorticoid indices (N = 112).

Variable	B	SE	Wald	df	Sig
CTQ	0.052	0.019	8.222	1	0.004
NR3C1	−3.317	1.449	5.242	1	0.022
FKBP5	0.838	0.346	5.879	1	0.015
KITLG	2.261	0.617	13.430	1	0 < 0.001

When CTQ score along with NR3C1, FKBP5 and KITLG levels were entered into the logistic regression model, they correctly classified 46 of 53 HY cases and 50 of 59 SY cases, resulting in 85.7% correct classification rate (Table 4).

**Table 4:** Correct classification rate of suicidal adolescents by Childhood trauma score and glucocorticoid indices.

	Healthy adolescents	Suicide adolescents	Percentage correct
Healthy adolescents	46	7	86.8%
Suicide adolescents	9	50	84.7%
Overall Percentage			85.7%

Linear regression analyses were applied to further evaluate the potential contribution of current salivary cortisol levels, NR3C1 and KITLG to current suicidal thoughts continuous score (as measured by the PHQ-9) derived from the entire adolescent cohort including the healthy controls. Salivary cortisol levels accounted for 10.4% of the score (p < 0.001 ß = −0.253, t = −2.838, p = 0.005). KITLG levels accounted for another 4% of the score (p = 0.024; ß=0.189, t = 2.145, p = 0.034) and NR3C1 levels accounted for another 3.3% of the score (p = 0.039; ß = −0.185, t = −2.093, p = 0.039). The entire model explains 17.7% of the variance of current suicidal ideation score (R = 0.421, F(3110) = 7.89, p < 0.001).

## Discussion

Compared with healthy adolescent controls, adolescents requiring hospital care following suicidal crises display higher exposure to childhood trauma and lower morning salivary cortisol levels along with altered expression of immune cell gene transcripts, including NR3C1 encoding the glucocorticoid receptor and its co-chaperons FKBP5 and KITLG when considered together using MANOVA, as well as when each variable is considered separately. The healthy control group was older and had a higher percentage of males compared with the suicidal group, however CTQ, salivary cortisol, and mononuclear cell gene expression levels of the three PBMC transcripts NR3C1, FKBP5, and KITLG, did not correlate with age, did not show significant mean differences between males and females among the entire sample, and mean differences in CTQ and biological indices between SY and HY remained significant following correction for age and gender as co-variates. Exposure to CT significantly correlates with each of the above glucocorticoid transcript levels. Taken together, CT scores, cortisol, and mononuclear cell glucocorticoid transcript levels, explain two-thirds of the variance in risk for being designated in the suicidal adolescent group, and cortisol and NR3C1 and KITLG levels together explain 17.7% of the variance in the PHQ-9 suicidal ideation score.

Childhood trauma has been repeatedly shown to constitute a strong risk factor for suicidality among adolescents [19], and adults [20–22] with evidence to suggest a dose–response effect [23]. Most previous studies found CT associates with a blunted cortisol stress response and lower basal cortisol [24–26]. Young depressed patients with non-suicidal self-injury exhibit lower cortisol levels and higher CT exposure [27], and likewise, bipolar patients with higher CT exposure had reduced cortisol awakening response as well as increased lifetime suicide attempts [28]. Previous studies further found CT levels positively correlated with the severity of suicidality, with CT predictive of blunted cortisol reactivity to stress and lower resting basal cortisol levels, and blunted stress reactivity predicting suicidality later in life [29, 30]. Adolescents and males showed more consistent associations, with age [31] and gender [32] likely adding to outcome variability in studies showing inconsistent results. Several previous studies found lower blood NR3C1 transcript levels among subjects with CT exposure [17, 33–36], although unchanged or increased levels have also been reported [33]. Lower baseline mononuclear NR3C1 transcript levels among healthy adolescents with higher CT scores were shown to predict blunted cortisol response to stress [17]. Altered brain and blood NR3C1 methylation and expression levels have been reported with CT-associated cortisol abnormalities and psychiatric sequel [33, 37]. In the current study design only morning salivary cortisol following hospitalization was sampled. Multiple sampling points that allow a comprehensive characterization of the entire diurnal cortisol dynamics would be more informative, as may be sampling of hair and nail cortisol which have been previously used to gain insight into cumulative cortisol dynamics over longer periods of time [38].

Lower prefrontal [39] and amygdalar [40] NR3C1gene expression were reported in completed suicide cases, and lower hippocampal [41, 42] NR3C1 gene expression was reported among completed suicide victims with a history of CT. Lower blood NR3C1 expression and lower hair cortisol were reported among youth following suicide attempt [43]. Lower mononuclear NR3C1 expression was found among suicidal depressed patients [44], and a genotype specific reduction in NR3C1 blood expression was reported in those with CT exposure [45]. The FKBP5 gene encodes a GR binding HSP-90 co-chaperone that reduces GR’s affinity to cortisol and their nuclear translocation, with activated GR inducing FKBP5 gene and protein expression, and FKBP5 intronic allelic variation and methylation status reported to alter GR sensitivity and cortisol regulation [46]. Transcript levels of FKBP5 were shown to be affected by genotype [47], and gene-environmental interaction with CT [48, 49], with previous studies reporting both CT-associated decreased [36] as well as increased [47, 50] blood FKBP5 transcript levels, and allele specific methylation status reported to modulate the effect of CT. Genetic variants in the FKBP5 gene were found to associate with suicide risk [39, 51–55], with FKBP5 genotype by CT interactions found to increase suicide risk [56, 57]. Lower amygdalar FKBP5 expression was reported among completed suicide cases [40], and lower blood mononuclear cell FKBP5 levels were reported to differentiate suicidal from non-suicidal depressed patients [44]. DNA methylation of the KITLG gene was previously reported to mediate the association between childhood trauma and cortisol stress reactivity, with higher KITLG blood methylation levels associating with lower levels of cortisol under stress [58, 59]. Associations of KITLG gene expression with CT and suicidality have not been explored to date.

Glucocorticoid treatment [60], as well as its withdrawal [61] have been associated with iatrogenic increases in suicidality among vulnerable subsets of patients, pointing to both the relevance and intricacy of these associations, which are likely shaped by individual susceptibility. We are not aware of a previous demonstration that altering the level of endogenous cortisol or the expression of the genes encoding the glucocorticoid receptor and its co-chaperons was associated with a prospective change in suicide risk. A chronic pro-inflammatory state has been repeatedly documented to follow CT [8], and inflammation has been suggested to contribute to suicide risk [62, 63]. Administration of pro-inflammatory cytokines including interleukine-1 (IL-1), interleukin-6 (IL-6), and tumor necrosis factor alpha (TNF-alpha) has been shown to induce HPA activation [64]. Compromised blood-brain-barrier permeability increasing CNS entry of peripheral cytokine, has been documented following suicide attempts and associated with increased cerebrospinal fluid (CSF) levels of the CD44 ligand hyaluronic acid, indicative of increased neuroinflammation [65]. Elevated CSF quinolinic acid was found following suicide attempts with lower kynurenic acid and higher IL-6 correlating with severity of suicidality and depression, suggesting underlying pro-inflammatory cytokine activation of indoleamine 2,3-dioxygenase and the kynurenine pathway [66]. Elevated CSF and post-mortem cortical brain levels of IL-1*β* and IL-6 have been reported to accompany suicidality among adults [67] and adolescents [68]. Glucocorticoids may thus exert their effects on suicidal tendencies in part through their action as potent modulators of the immune inflammatory response. Altered in vivo dexamethasone suppression of cortisol secretion has been associated with childhood trauma [69, 70], and demonstrated to predict suicidality among adolescents [71, 72] and adults [73, 74], with persisting alterations after recovery from depression differentiating those with recurrent suicide attempts [75]. In vitro sensitivity to dexamethasone suppression of proinflammatory activation of mononuclear subsets has been previously shown to predict psychopathological outcomes following stressful exposure, suggesting glucocorticoid receptor sensitivity (GCRS) may represent a persistent biological vulnerability factor for stress related conditions [76]. Modulators of FKBP5 gene function have been shown to counteract the effects of its encoded immunophilin FK506-binding-protein-5 on macrophage polarization [77] as well as on trauma-related behaviors and corticosterone levels in preclinical models [78], supporting a therapeutic potential for such approach. Correlating individual immune cell glucocorticoid pathway gene expression levels with alterations in mononuclear cell culture GCRS regulation of immune activation, can be employed to guide systematic experimental modulation of GCRS through in vitro manipulation of FKBP5 and KITLG function, to explore a therapeutic potential for an individually tailored immune modulation approach based on immune cell transcriptional signatures. Specific transcriptional alterations serving as biomarkers of abnormal immune glucocorticoid reactivity could potentially help guide future individually tailored application of selective glucocorticoid receptor modulators [79] to fine-tune empirically defined pathological stress reactivity aimed at reducing CT-associated pro-inflammatory burden with its adverse cardiovascular consequence [80] and perhaps also affecting CNS psychopathology.

Our results support a compound biopsychosocial susceptibility model whereby higher CT exposure along with altered cortisol and transcriptional correlates of immune cell glucocorticoid reactivity associate with clinically significant suicidal crises during the sensitive window of adolescence. The Childhood Trauma Questionnaire (CTQ) [13] probes cumulative adversity exposure prior to age 18. All study participants underwent comprehensive structured clinical interviews and none reported acute exposure to traumatic events fulfilling DSM-V A criterion for acute stress disorder or acute PTSD in the months prior to presentation.

While CT explains a part of the variance of current adolescent suicidality and correlates with key mononuclear cell glucocorticoid transcript expression levels, salivary cortisol levels together with glucocorticoid transcript expression levels contribute a large portion of the variance beyond that accounted for by CT. Within such susceptibility model, the lack of baseline sampling points prior to trauma exposure in childhood, as well as to later suicidal crises, preclude resolving the origination of implicated compromise in hormonal-immune stress response. Earlier sampling points are requisite to prospectively address whether cortisol and transcriptional profiles observed in adolescence may be consequent to or precede CT or suicidality. Conceptually, a compromised hormonal-immune stress response could either precede and predispose to psychopathological responses to adverse exposures at both childhood and adolescence time points, or alternatively either follow, or become further sensitized, consequent to early adverse exposure. While early life social adversity has been shown to reprogram offspring DNA methylation and later life behavioral and endocrine – immune stress reactivity, advancing one putative mechanism [81, 82], longitudinal studies have suggested a complex pattern of causality whereby familial and socioeconomic factors preceding childhood victimization account for cognitive deficits that may contribute to children’s vulnerability to victimization [83]. A study employing Mendelian Randomization analyses (MRA) to disentangle and extract causal inferences between reported childhood trauma and major depressive disorder (MDD), suggested that the experience of traumatic events can increase the risk for MDD, but that MDD can increase the experience of traumatic events [84]. Decreased baseline pre-deployment blood FKBP5 transcript levels [85] and higher C-reactive protein levels [86] were found to predict increased risk for post-traumatic symptoms following combat deployment among large cohorts of deployed combat soldiers. As our study, along with most of the literature, can detect correlation based on a cross-sectional perspective rather than prospectively deciphering causation, we refrained from attempting to regress CTQ with transcriptional data, for lack of an unequivocal hypothesis to guide the direction of the regression one way or the other.

Although a large portion of the variance in suicidal group assignment was explained by CT and glucocorticoid indices in the current sample, replication, as well as additional risk factors, are required for constructing a clinically useful risk model, and longitudinal follow-up studies are needed to determine their utility for prospective risk prediction. Despite these limitations, our results demonstrate a realistic empirical model for approaching a complex phenomenon such as clinically significant adolescent suicidality by incrementally applying relevant risk factors and, perhaps more importantly, point to potentially modifiable risk factors for focusing individually tailored preventive psychosocial and biological intervention efforts based on empirically characterized vulnerability factors. One way of validating a causal role for CT and glucocorticoid indices in contributing to clinically significant suicidality would be to guide the construction of future prospective interventions to measure if addressing empirically identified risk factors would provide a clinically significant reduction in prospectively assessed subsequent suicide risk.

## Data availbability

The datasets generated and/or analysed during the current study are available from the corresponding author on reasonable request.

## Supplementary information

Goltser et al Supplementary Information
