# Supplementary material for: Childhood trauma cortisol and immune cell glucocorticoid transcript levels are associated with increased risk for suicidality in adolescence
Source: Mol Psychiatry. 2025 Feb 24;30(8):3376–83. doi: 10.1038/s41380-025-02923-3 (PMC12240822; doi:10.1038/s41380-025-02923-3)
Supplement: Supplementary file 1 — Goltser et al Supplementary Information [file 41380_2025_2923_MOESM1_ESM.docx]

**Supplementary Fig 1. Childhood trauma scores and mononuclear cell transcript levels among suicidal *vs.* healthy control adolescents**


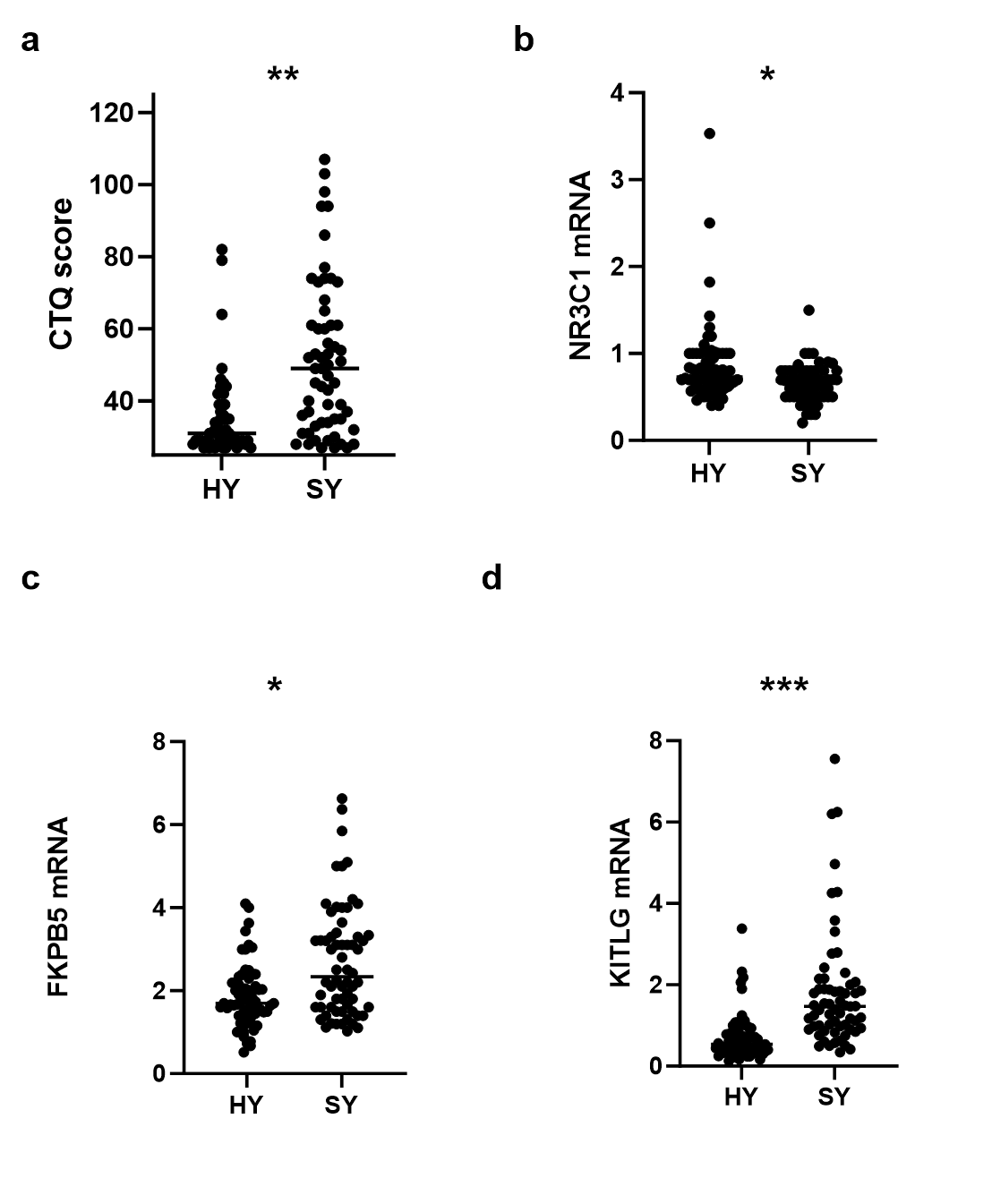


**Supplementary Fig 1.** The correlation graphs depict Childhood Trauma Questionnaire (CTQ) scores and mononuclear cell glucocorticoid transcript levels, for the two groups Healthy Youth (HY) and Suicidal Youth (SY). CTQ SY (59) vs. HY (53) B=.052, SE=.019 p=.004 (Fig3a); NR3C1 SY (*n*=63) *vs.* HY (*n=*69) B=-3.117, SE=1.449 p=.022 (Fig. 3b); FKBP5 SY (*n*=63) *vs.* HY (*n=*69) B=.838, SE=.346, p=015 (Fig3c); KITLG SY (*n*=63) *vs.* HY (*n=*69) B=2.61, SE=.617, p<.001 (Fig3d); *= p≤.05, **= p≤.01, ***=p≤.001
